# Role of Serum Ferritin in Predicting Outcomes of COVID-19 Infection Among Sickle Cell Disease Patients: A Systematic Review and Meta-Analysis

**DOI:** 10.3389/fmed.2022.919159

**Published:** 2022-05-30

**Authors:** Jun Xin Lee, Wei Keong Chieng, Muhammad Irfan Abdul Jalal, Chai Eng Tan, Sie Chong Doris Lau

**Affiliations:** ^1^Department of Pediatrics, Faculty of Medicine, Universiti Kebangsaan Malaysia, Kuala Lumpur, Malaysia; ^2^Department of Family Medicine, Faculty of Medicine, Universiti Kebangsaan Malaysia, Kuala Lumpur, Malaysia; ^3^UKM Medical Molecular Biology Institute, Universiti Kebangsaan Malaysia Medical Centre, Kuala Lumpur, Malaysia

**Keywords:** sickle cell disease, COVID-19, ferritin, ICU, mortality

## Abstract

Patients with sickle cell disease (SCD) are at higher risk of getting severe COVID-19 infection. This systematic review and meta-analysis aimed to determine the role of serum ferritin in predicting ICU admission and mortality among patients with SCD following COVID-19 infection. A systematic search was conducted in PubMed, Scopus, Web of Science, Embase, WHO COVID-19 database, ProQuest, and Cochrane Library for articles published between 1st December 2019 to 31st November 2021. Methodological quality was assessed using the Joanna Briggs Institute (JBI) critical appraisal checklists. Eleven articles (7 cohorts and 4 case series) were included in this review. Pooled mean serum ferritin level on admission was 1581.62 ng/mL while pooled proportion of ICU admission and mortality were 0.10 (95% CI 0.06; 0.16, prediction interval 0.04; 0.23, *p* = 0.29, *I*^2^ = 17%) and 0.07 (95% CI 0.05; 0.11, prediction interval 0.04; 0.12, *p* = 0.68, *I*^2^ = 0%) respectively. Meta-regression showed that serum ferritin did not predict for both ICU admission (regression coefficient = 0.0001, *p* = 0.3523) and mortality (regression coefficient = 0.0001, *p* = 0.4029). Our analyses showed that serum ferritin may not be a useful marker to predict the outcomes of COVID-19 infection among patients with SCD. More data are required to identify a reliable tool to identify patients with SCD who are at risk of getting severe COVID-19 infection.

**Systematic Review Registration:**

https://www.crd.york.ac.uk/prospero/display_record.php?RecordID=287792, PROSPERO Registration: CRD42021287792.

## Introduction

By the end of year 2021, global reported deaths from COVID-19 pandemic have reached a devastating number of 5.9 million (1). Patients with chronic illness such as chronic kidney disease, chronic respiratory or cardiovascular disease, diabetes mellitus, and hypertension were identified as high-risk groups with 2–8 fold increased risk of mortality compared to general population (2). Published data have identified several clinical and laboratory parameters which were useful to predict the outcomes of COVID-19 infection (3). High serum ferritin has been associated with immune dysregulation and cytokine storm in severe COVID-19 infection, and thus has been reported to be a useful tool to predict the disease severity in the general population (4–6).

Sickle cell disease (SCD) is an inherited blood disorder characterized by chronic anemia, acute painful crisis and organ infarction (7). International data revealed 20–25 million individuals worldwide have homozygous SCD, majority of which resides in sub-Saharan Africa and India (8). High risk patients with SCD require chronic exchange or blood transfusion as part of their treatment protocol, leading to iron overload and its multiple complications (9). Several studies reported an increased risk of severe COVID-19 infection and mortality among patients with SCD (10–13). However, with the existing chronic iron overload in this cohort of patients, we hypothesized that in patients with SCD, serum ferritin may not be a useful outcome predictor for COVID-19 infection.

This systematic review aimed to determine the role of serum ferritin in predicting outcomes of COVID-19 infection among patients with SCD. The outcomes of interest are intensive care unit (ICU) admission and mortality.

## Materials and Methods

### Study Protocol and Guideline

This systematic review and meta-analyses were conducted in accordance with the Preferred Reporting Items for Systematic Reviews and Meta-Analyses (PRISMA) guidelines (14). For data that could not be meta-analyzed, the findings from narrative synthesis were reported using the Synthesis without meta-analysis (SWiM) guideline (15). The study protocol was registered in the International Prospective Register of Systematic Reviews (PROSPERO) (Protocol number #CRD42021287792).

### Search Strategy and Selection Criteria

Systematic searches were performed in the following databases: PubMed, Scopus, Web of Science, Embase, WHO COVID-19 database, ProQuest Dissertations, and Theses Global and Cochrane Library. The combinations of search terms were #1: “COVID-19” [MeSH] AND “Anemia, Sickle Cell [MeSH]; #2: “Covid-19 [MeSH]” AND “Anemia, Sickle Cell [MeSH] AND “Ferritins [MeSH]; #3: “Covid-19 [MeSH]” AND “Anemia, Sickle Cell [MeSH] AND “Ferritins [MeSH] OR “Predictor” OR “Prognostic factor”; #4: “Covid-19 [MeSH]” AND “Anemia, Sickle Cell [MeSH] AND “Ferritins [MeSH] OR “Survival.” Manual searching was also conducted to identify potential articles from the reference list of included articles. The last search was conducted on the 30th November 2021.

Studies extracted from the searches were identified by two independent reviewers. All citation records were managed with EndNote(R) version 20 (The EndNote Team, Philadelphia, USA) and duplications were removed. The authors independently screened for relevant articles by analyzing the research title, abstract and index terms of the manuscripts. Studies that reported sufficient estimates of clinical parameters of interest and COVID-19 outcomes among SCD patients with the following study designs were included: systematic reviews, cohort studies, case control studies including nested case control studies, analytical cross-sectional studies, and case series. Case reports, editorials, opinion pieces, articles that reported suspected COVID-19 cases without laboratory evidence, *in vitro* studies and patients without SCD were excluded from this review. For published articles that reported data obtained from overlapping populations, only the most recent publication was selected.

### Data Collection and Methodological Quality Assessment

Full-text articles fulfilling inclusion criteria were retrieved to assess their eligibility. For studies that reported laboratory parameters without serum ferritin, the authors attempted to contact the corresponding authors to obtain the raw data, failing which these studies were excluded. The included citations were exported to an Excel spreadsheet and coded as following: author/s, study title, study design, country of study, year of publication, sample size, age, gender, comorbidities, current treatment with hydroxyurea, serum ferritin, ICU admission and mortality. Serum ferritin was presented as means and standard deviations, using reported values or estimated using standard formulas for studies that reported median and interquartile range (16). The data extracted from each article was cross-checked by at least two independent investigators. An independent third reviewer evaluated the data and provided the final decision in the event of any dispute.

The methodological quality of studies included was assessed using the Joanna Briggs Institute (JBI) critical appraisal tools for the respective study designs (17). The studies were further classified into poor (0–49%), moderate (50–69%), or high (70% and above) quality (18, 19). The risk of bias was assessed independently by two investigators and any discrepancies in opinions were resolved by a third investigator.

### Operational Definitions

In this review, serum ferritin referred to the ferritin level during admission for COVID-19 infection. Comorbidities were categorized based on respective systems namely cardiovascular, respiratory, endocrine, renal, oncology, musculoskeletal, and others. History of acute chest syndrome (ACS), vaso occlusive crisis, splenectomy, and transfusion frequency were also recorded. Outcomes of COVID-19 infection were determined by ICU admission and mortality rate. ICU admission signified severe (at least Category 4) COVID-19 infection.

### Statistical Analysis

R packages, metafor version 3.0, meta, and dmetar were used for meta-analysis and meta-regression (20, 21). Both packages were implemented in R version 4.1.1 (R Core Team, Vienna, Austria) using RStudio version 1.4.1106 (RStudio Team, Boston, USA).

Using random effects models, pooled means, and standard deviations for serum ferritin and pooled proportions with logit transformation (22) for severity and mortality rate of COVID-19 infection in patients with SCD were computed. Restricted Maximum Likelihood (REML) and the Hartung-Knapp adjustments were used to estimate the variances, τ2 for both effect measures and to calculate the confidence intervals of summary effects, respectively. The 95% confidence interval for τ2 was obtained using the Q-profile method.

Heterogeneity was assessed using the Cochran χ^2^-test (*p* <0.1 indicates significant heterogeneity), and the *I*^2^ statistic which was classified as following: (i) 0–40%: possibly unimportant; (ii) 30–60%: moderate heterogeneity; (iii) 50–90%: substantial heterogeneity, and (iv) 75–100%: considerable heterogeneity (23). Subgroup and sensitivity analyses were performed in the presence of substantial heterogeneity (Cochran χ^2^ *p* < 0.1 or *I*^2^ > 50%).

Publication bias was evaluated by subjective visual inspection of the funnel plots for the lack of small-size studies with small effect size and objectively by Egger's test. The Galbraith plot was used to detect the presence of outlier studies (24).

Serum ferritin was evaluated as a predictor for COVID-19 outcomes among patients with SCD using meta-regression. All tests were two-tailed, and *p*-values of 0.05 or lower were considered to be statistically significant.

## Results

### Systematic Search Results

**Figure 1:**
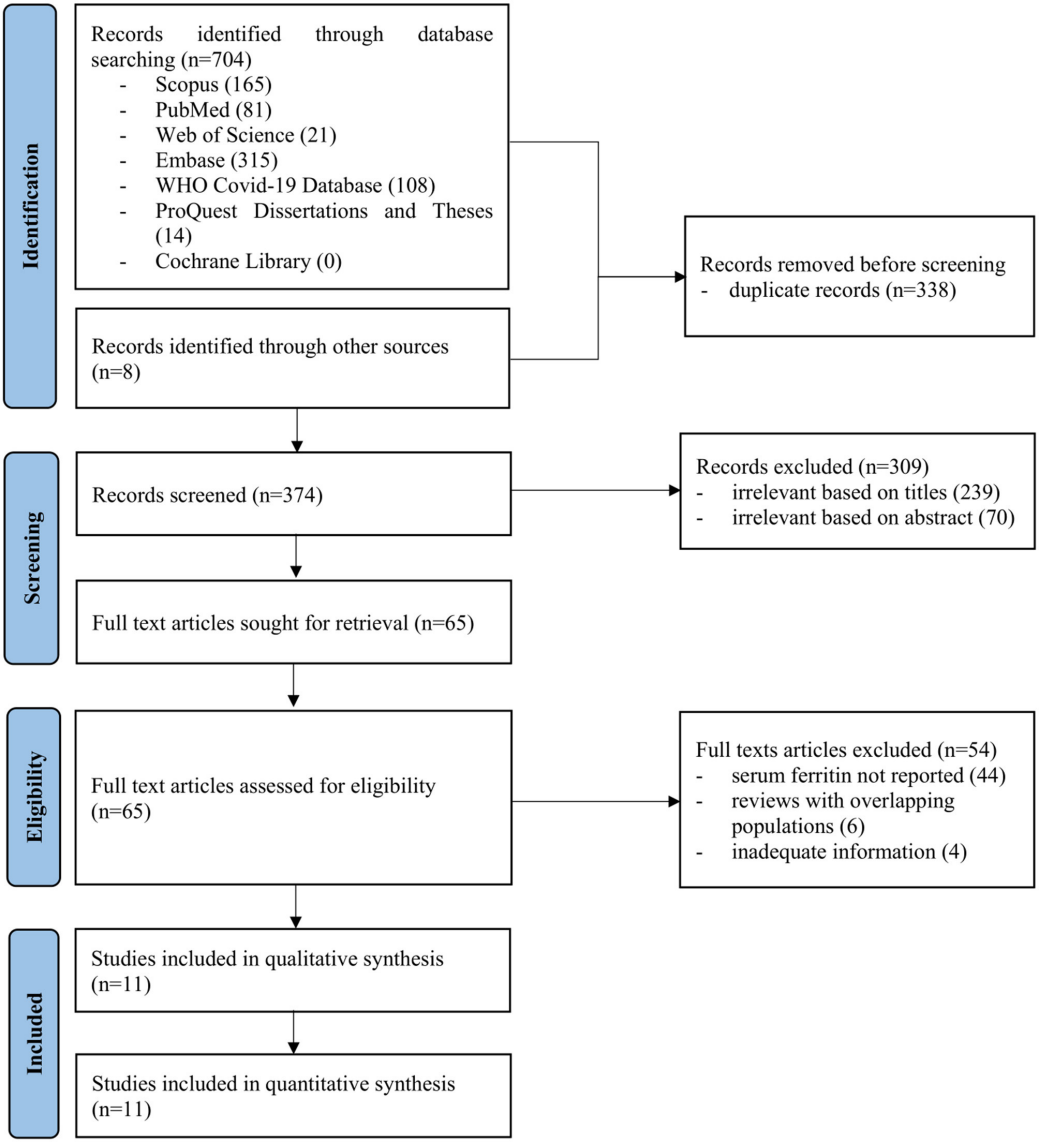
Flow chart of study selection process (flow chart adapted from PRISMA).

The searches of electronic databases yielded 704 articles while manual search through the references of these articles contributed additional 8 articles (Figure 1). Overall, 374 titles and abstracts were screened after removing 338 duplicates. Following this, 65 full-text articles were retrieved and reviewed. Eleven studies which consisted of 7 cohorts and 4 case series were finally included in this review (Table 1). The methodological quality for all the studies were summarized in the Supplementary Table 1.

**Table 1:** Characteristics of studies included.

**No**.	**Reference**	**Study type**	**Country**	**N**	**Age in years, mean (range)**	**Male, *n***	**Ferritin (ng/mL) on admission, mean (SD)**	**ICU admission, *n***	**Mortality, *n***
1.	Alhumaid et al. (25)	Cohort	Saudi Arabia	31	NR	NR	965 (617)	14	NR
2.	Alkindi et al. (26)	Cohort	Saudi Arabia	50	31^*^	27	1,829 (78)	2	2
3.	Anusim et al. (27)	Case series	United States	11	44 (22-60)	4	5,571 (4,110)	2	2
4.	Balanchivadze et al. (28)	Case series	United States	24	53 (24-87)	6	820 (494)	1	1
5.	Boga et al. (29)	Cohort	Turkey	39	35 (18–64)	17	847 (908)	4	2
6.	Devarashetty et al. (30)	Cohort	United States	51	30^*^	20	787 (544)	2	2
7.	McCloskey et al. (31)	Case series	United Kingdom	10	36 (23–57)	8	4,669 (6,123)	0	1
8.	Minniti et al. (13)	Cohort	United States	66	33 (24-40)	30	1,035 (615)	6	7
9.	Ramachandran et al. (32)	Case series	United States	9	28 (19-40)	5	2,026 (1,550)	1	0
10.	Sewaralthahab et al. (33)	Cohort	United States	21	42^*^	5	1,141 (857)	5	2
11.	Yurtsever et al. (34)	Cohort	United States	40	30 (2–66)	17	4,166 (7,730)	6	1

*N, number; SD, standard deviation; ICU, intensive care unit*.

* *Studies reporting age in median*.

Overall, a total of 352 patients with SCD contracted COVID-19 infection. The patients' mean age was 34.5 years (range from 2 to 87 years old) with male patients making up 43.3% (*n* = 139) of the cohort. Only one paper published data on 13 pediatric patients. The most common genotype reported was HbSS genotype (*n* = 159, 45.2%) followed by HbS/thalassemia (*n* = 65, 18.5%). Approximately half of the patients (*n* = 187, 58.3%) did not have any comorbidities (Supplementary Table 2). One-third (*n* = 122; 38.0%) of the patients were on hydroxyurea therapy while 45 patients (14.0%) were on regular blood transfusion. None of the studies reported on the patients' COVID-19 vaccination status.

### Serum Ferritin on Admission for COVID-19 Infection

**Figure 2:**
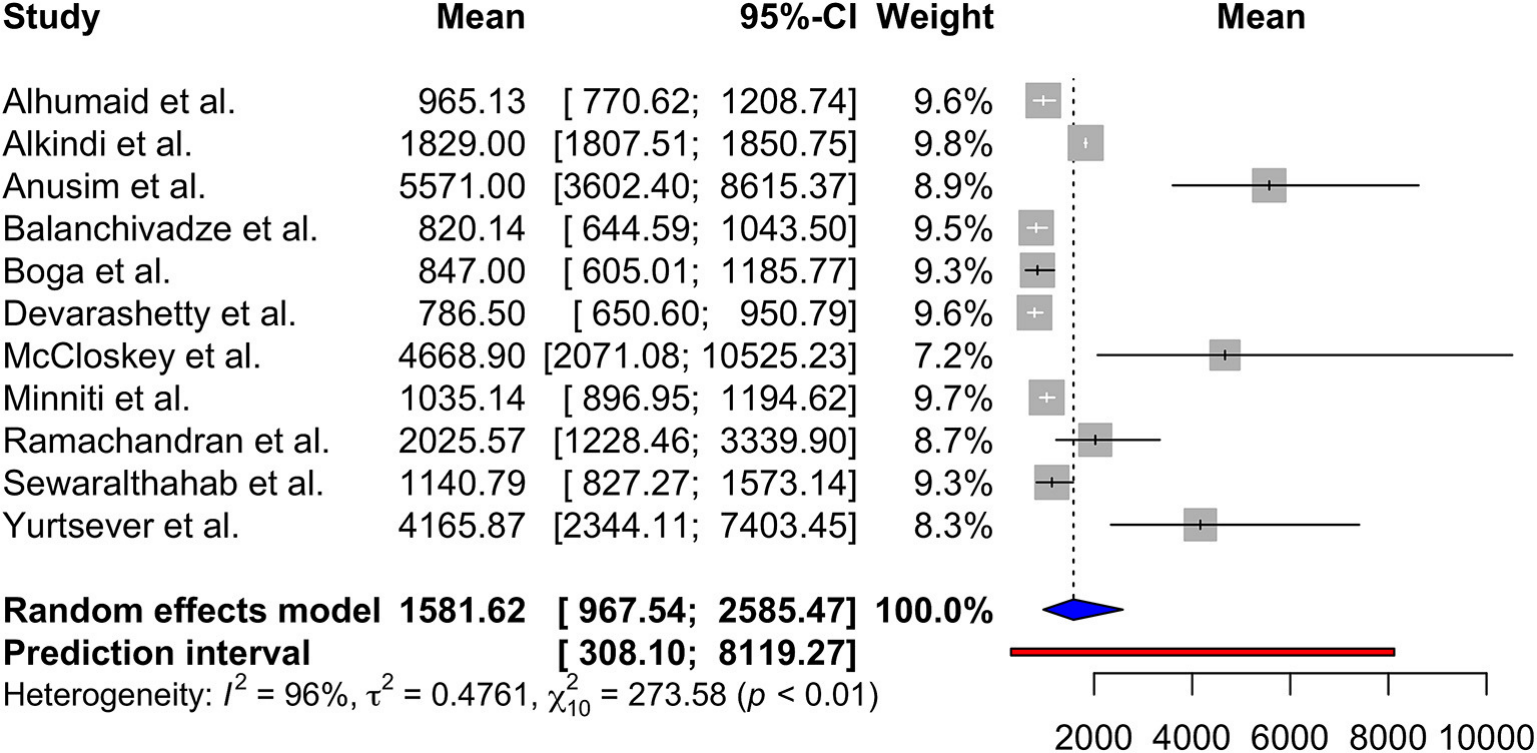
Forest plot showing mean serum ferritin (ng/mL) on admission.

Using random effects model, the pooled mean serum ferritin was 1581.62 ng/mL (95% CI 967.54; 2585.47, prediction interval 308.10; 8119.27, *p* < 0.01). There was substantial between-study heterogeneity (*I*^2^ = 96%) (Figure 2).

### COVID-19 Outcomes in Patients With SCD

**Figure 3:**
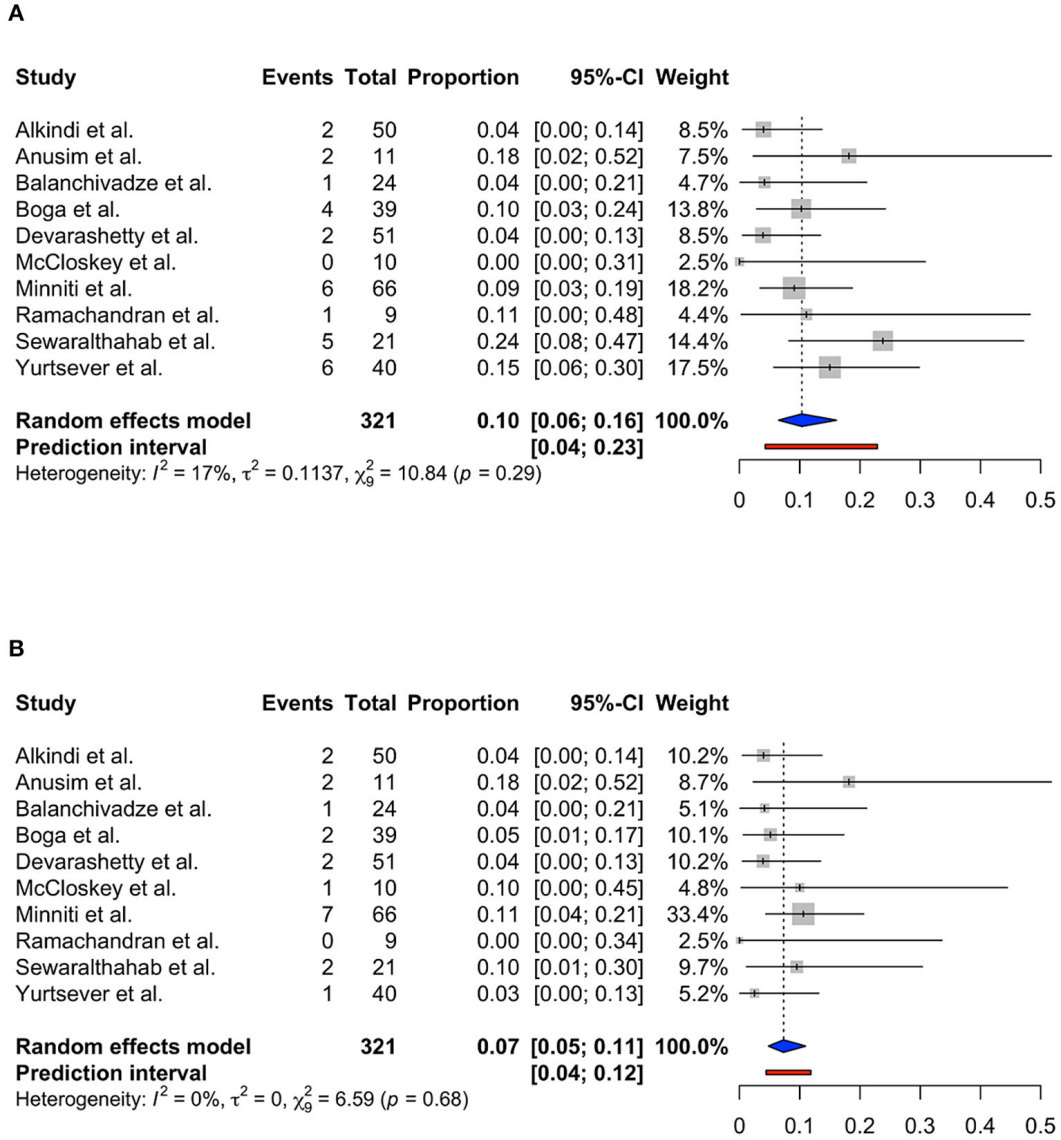
Forest plot showing **(A)** ICU admission and **(B)** mortality due to COVID-19 infection.

Only 10 studies were included into the meta-analysis to determine the role of serum ferritin as a predictor for ICU admission and mortality among patients with SCD. Study by Alhumaid et al. was not included as the data required for meta-analysis was insufficient (25). Using the random effects model, the pooled proportion of ICU admission was 0.10 (95% CI 0.06; 0.16, prediction interval 0.04; 0.23, *p* = 0.29) with low heterogeneity (*I*^2^ = 17%) (Figure 3A). The pooled proportion of COVID-19 mortality in patients with SCD was 0.07 (95% CI 0.05; 0.11, prediction interval 0.04; 0.12, *p* = 0.68) with no heterogeneity (*I*^2^ = 0%) (Figure 3B).

**Figure 4:**
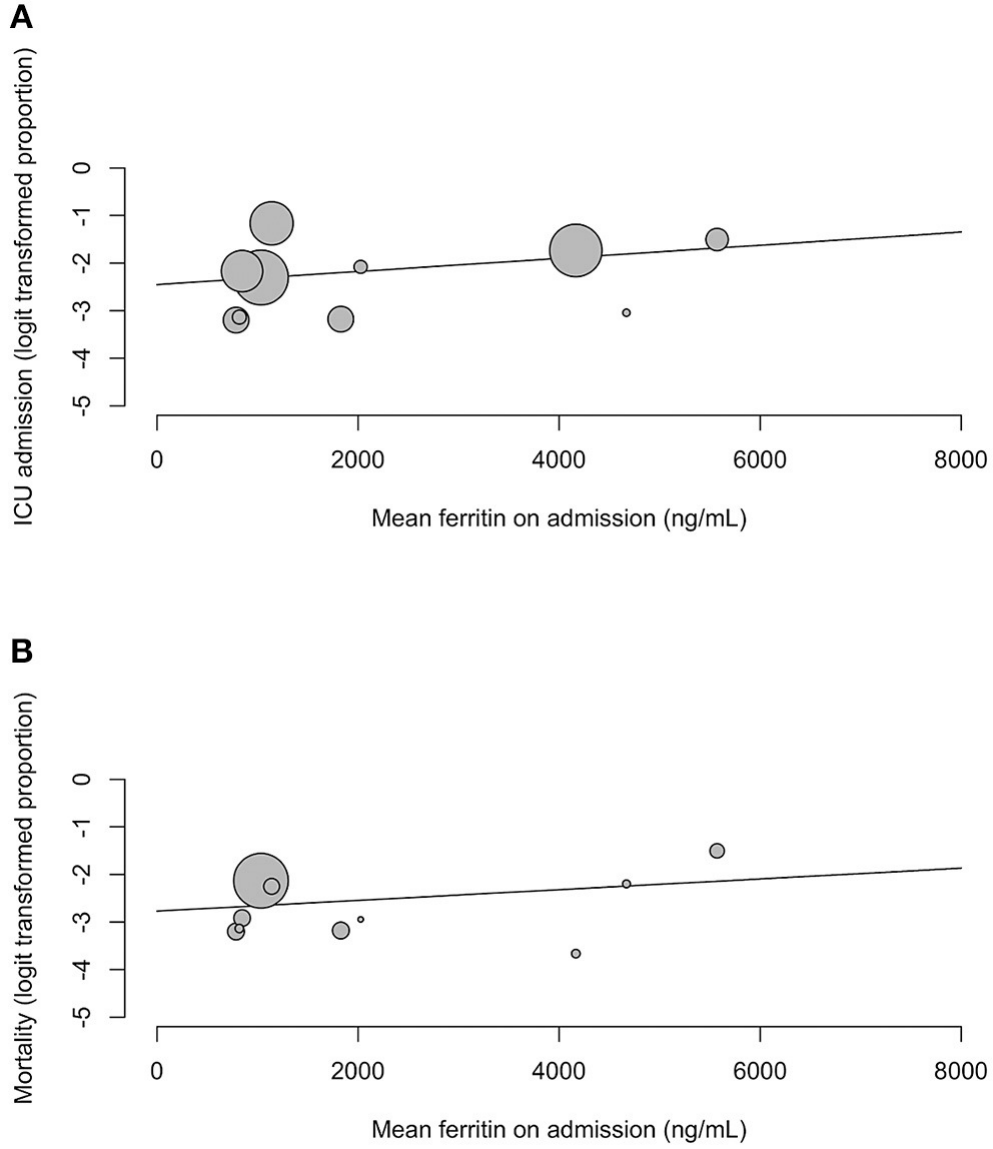
Meta-regression bubble plots for **(A)** ICU admission and **(B)** mortality against mean serum ferritin on admission.

Our meta-regression showed that serum ferritin on admission for COVID-19 did not predict for ICU admissions (regression coefficient = 0.0001, OR = 1, 95% CI 1.00; 1.00, *p* = 0.3523) and mortality (regression coefficient = 0.0001, OR = 1, 95% CI 1.00; 1.00, *p* = 0.4029) (Figure 4). Further subgroup analysis and meta-regression revealed no significant associations between serum ferritin and study characteristics, sociodemographic factors or underlying comorbidities.

### Publication Bias

The funnel plots and Egger's test showed no publication bias for serum ferritin (*p* = 0.1532), ICU admission (*p* = 0.2099) and mortality (*p* = 0.1925) (Supplementary Figure 1). Galbraith plots showed no outlier studies were detected in the meta-analysis (Supplementary Figure 2).

## Discussion

Our study showed that serum ferritin levels on admission for COVID-19 infection did not predict ICU admission and mortality among patients with SCD. The role of serum ferritin in predicting severity and mortality of COVID-19 infection has been inconsistent in other studies. Two previous meta-analyses done on general population reported significantly higher levels of serum ferritin among patients with severe COVID-19 and among those who succumbed (4, 35). The heterogeneity among these studies and publication bias were, however, significant and no risk ratio was reported to suggest the directionality of ferritin in predicting severity and mortality of COVID-19 infection. Conversely, other studies demonstrated that although elevation of serum ferritin to over 25th percentile had significantly higher odds of more severe lung involvement (36), serum ferritin was neither associated with COVID-19 severity (36–38) nor mortality (36, 37). Our findings concurred that serum ferritin did not predict COVID-19 severity or mortality among patients with SCD.

Ferritin is an acute phase reactant, as well as a mediator of immune dysregulation during cytokine storms in severe COVID-19 infection (39). Inflammatory cytokines such as interleukins, tumor necrosis factors, and interferons are rapidly secreted during cytokine storms, which in turn upregulate the production of ferritin by hepatocytes, Kupffer cells, and macrophages (40). Concurrently, ferritin also induces the release of pro-inflammatory and anti-inflammatory cytokines (36). The role of ferritin in the cytokine storm explained its role as a predictor for poor outcomes in various conditions (41–44). However, our review found that it did not predict for ICU admission or mortality among patients with SCD who were infected with COVID-19. One possible explanation for this could be the central role of chronic inflammation in the pathophysiology of SCD (45, 46). This ongoing inflammatory process leds to a higher baseline serum ferritin among patients with SCD compared to the general population, and further elevation happens during acute painful crises (47–49). Frequent blood transfusions, shorter duration of red cells survival and chronic intravascular hemolysis further contribute to a significantly higher levels of ferritin in these patients (50, 51). Therefore, it is possible that the higher baseline serum ferritin level in patients with SCD may have diminished its ability to predict the outcome of COVID-19 infection.

This meta-analysis provided evidence that serum ferritin may not be a useful marker to predict the outcomes for COVID-19 infection among patients with SCD with regards to ICU admission and mortality. However, our review is limited by the small number of eligible studies for meta-analysis, and insufficient reported details such as stratification of serum ferritin levels by disease severity and clinical outcomes. High between-study effect heterogeneity for the serum ferritin results was also observed, although our sensitivity analysis revealed no “outlying” studies which may have led to this (Supplementary Figure 3). Therefore, we postulate that this high heterogeneity could be contributed by other factors such as time-point of ferritin measurement after admission, varying laboratory analyzers to determine serum ferritin level, and the differences in the study population's disease control. Hence, our findings need to be interpreted with caution.

## Conclusion

Our study showed that serum ferritin level on admission for COVID-19 infection did not predict the risk for ICU admission and mortality among patients with SCD. More data are required to identify other reliable clinical and laboratory markers as COVID-19 outcome predictors in this group of patients.

## Data Availability Statement

The original contributions presented in the study are included in the article/Supplementary Material, further inquiries can be directed to the corresponding author/s.

## Author Contributions

JL, WC, MA, CT, and SL contributed to the conception and design of the study. JL and WC conducted the systematic search of the study. JL, WC, and MA contributed to the data analysis. All authors contributed to the drafting and revising the article and gave final approval of the version for publication.

## Conflict of Interest

The authors declare that the research was conducted in the absence of any commercial or financial relationships that could be construed as a potential conflict of interest.

## Publisher's Note

All claims expressed in this article are solely those of the authors and do not necessarily represent those of their affiliated organizations, or those of the publisher, the editors and the reviewers. Any product that may be evaluated in this article, or claim that may be made by its manufacturer, is not guaranteed or endorsed by the publisher.
