# Supplementary material for: Role of Serum Ferritin in Predicting Outcomes of COVID-19 Infection Among Sickle Cell Disease Patients: A Systematic Review and Meta-Analysis
Source: Front Med (Lausanne). 2022 May 30;9:919159. doi: 10.3389/fmed.2022.919159 (PMC9196080; doi:10.3389/fmed.2022.919159)
Supplement: Supplementary file 1 [file Data_Sheet_1.docx]

Supplementary Material

Supplementary Table 1. Methodological quality of included studies based on the JBI critical appraisal tools for each study design.

| Author | Study Design | Item  1 | Item  2 | Item  3 | Item  4 | Item  5 | Item  6 | Item  7 | Item  8 | Item  9 | Item 10 | Item 11 | Quality |
| --- | --- | --- | --- | --- | --- | --- | --- | --- | --- | --- | --- | --- | --- |
| Alhumaid | Cohort | *√* | *√* | *√* | *√* | ↔ | *√* | *√* | NA | NA | NA | *√* | Mod |
| Alkindi | Cohort | *√* | *√* | *√* | *√* | X | *√* | *√* | *√* | NA | NA | *√* | Good |
| Boga | Cohort | *√* | *√* | *√* | ↔ | X | *√* | *√* | *√* | *√* | X | *√* | Good |
| Devarashetty | Cohort | *√* | *√* | *√* | X | X | *√* | *√* | NA | NA | NA | *√* | Mod |
| Minniti | Cohort | *√* | *√* | *√* | *√* | *√* | *√* | *√* | *√* | *√* | *√* | *√* | Good |
| Sewaralthahab | Cohort | *√* | *√* | *√* | X | X | *√* | *√* | NA | NA | NA | *√* | Mod |
| Yurtsever | Cohort | *√* | *√* | *√* | *√* | *√* | *√* | *√* | *√* | NA | NA | *√* | Good |
| Anusim | Case Series | ↔ | *√* | ↔ | *√* | *√* | *√* | *√* | *√* | *√* | NA | - | Good |
| Balanchivadze | Case Series | *√* | *√* | *√* | *√* | *√* | *√* | *√* | *√* | *√* | NA | - | Good |
| McCloskey | Case Series | *√* | *√* | *√* | *√* | *√* | *√* | *√* | *√* | ↔ | NA | - | Good |
| Ramachandran | Case Series | *√* | *√* | *√* | *√* | *√* | *√* | *√* | *√* | *√* | NA | - | Good |

*√, Yes; X, No; ↔, Unclear; NA, not applicable*

Good: 70% and above

Moderate: 50-69%

Poor: 0-49%

**Supplementary Table 2.** Underlying comorbidities in patients with SCD admitted for COVID-19 infection.

| **Underlying Medical Conditions [N=321]** | **n (%)** |
| --- | --- |
| None | 187 (58.3) |
| **SCD-related** | |
| History of acute chest syndrome / vaso occlusive crises | 134 (41.7) |
| History of splenectomy | 35 (10.9) |
| **Cardiology** | |
| Pulmonary hypertension | 20 (6.2) |
| Hypertension | 19 (5.9) |
| Congestive heart failure | 10 (3.1) |
| Coronary artery disease | 1 (0.3) |
| Aortic coarctation | 1 (0.3) |
| Valvular heart disease | 1 (0.3) |
| Arrhythmia | 1 (0.3) |
| Unspecified | 2 (0.6) |
| **Respiratory** | |
| Asthma | 13 (4.0) |
| Chronic obstructive pulmonary disease | 3 (0.9) |
| Pulmonary embolism | 2 (0.6) |
| **Neurology** | |
| Stroke | 33 (10.3) |
| Transient ischemic attack | 15 (4.7) |
| Trigeminal neuralgia | 1 (0.3) |
| **Renal** | |
| End stage renal disease / chronic kidney disease | 36 (11.2) |
| **Endocrine** | |
| Diabetes | 15 (4.7) |
| Obesity | 16 (5.0) |
| **Musculoskeletal** | |
| Joint necrosis | 41 (12.8) |
| **Hepatobiliary** | |
| Gallstone disease | 16 (5.0) |
| Chronic liver disease | 11 (3.4) |
| **Vascular** | |
| Venous thromboembolism | 35 (10.9) |
| **Oncology** | |
| Malignancy | 5 (1.6) |
| **Ophthalmology** | |
| Sickle cell retinopathy | 7 (2.2) |

*n, number; SCD, sickle cell disease*

**Supplementary Figure 1.** Funnel plots of (A) ICU admission, (B) mortality and (C) serum ferritin on admission for COVID-19 infection.


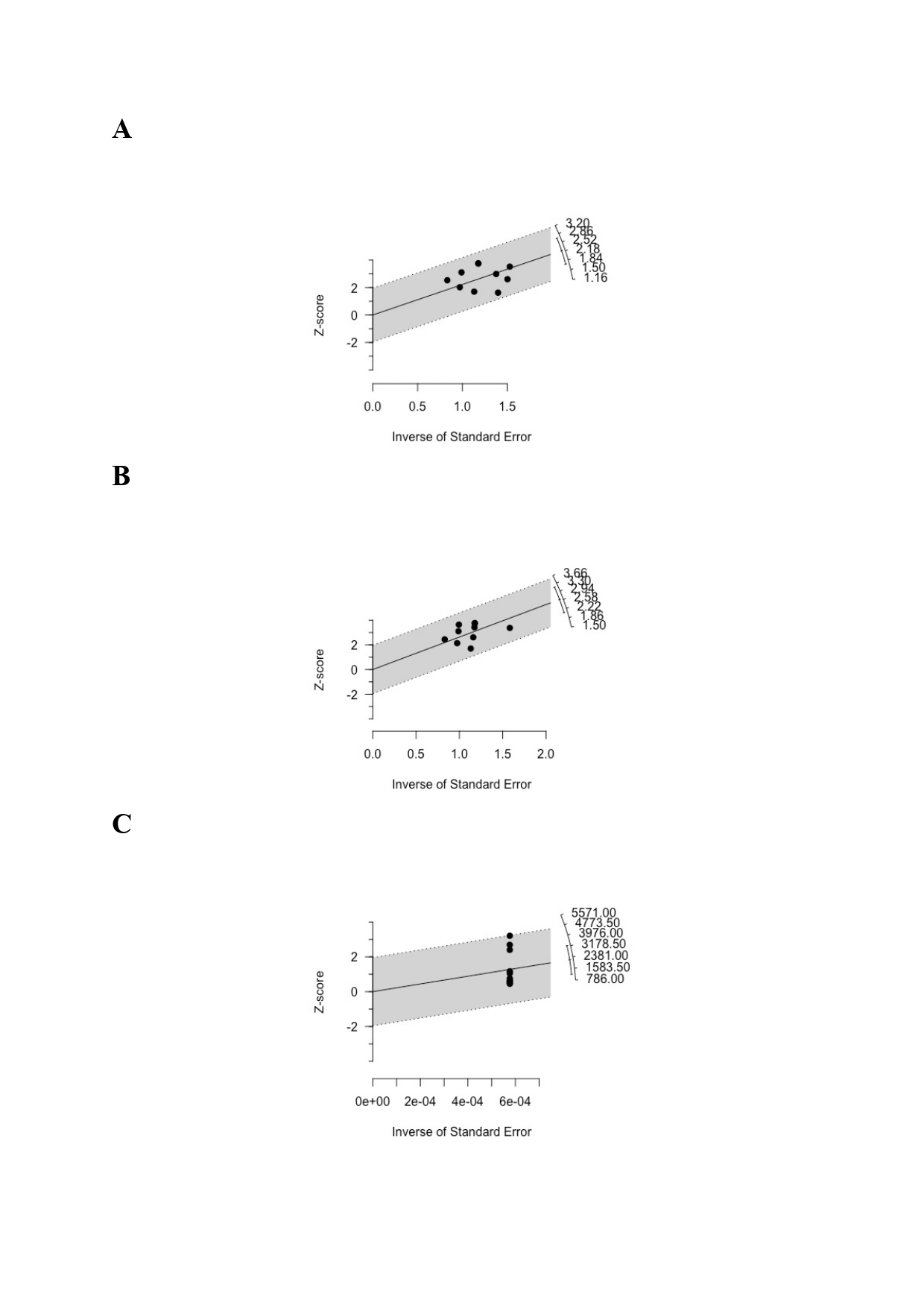
**Supplementary Figure 2.** Galbraith plots of (A) ICU admission, (B) mortality and (C) serum ferritin on admission for COVID-19 infection.


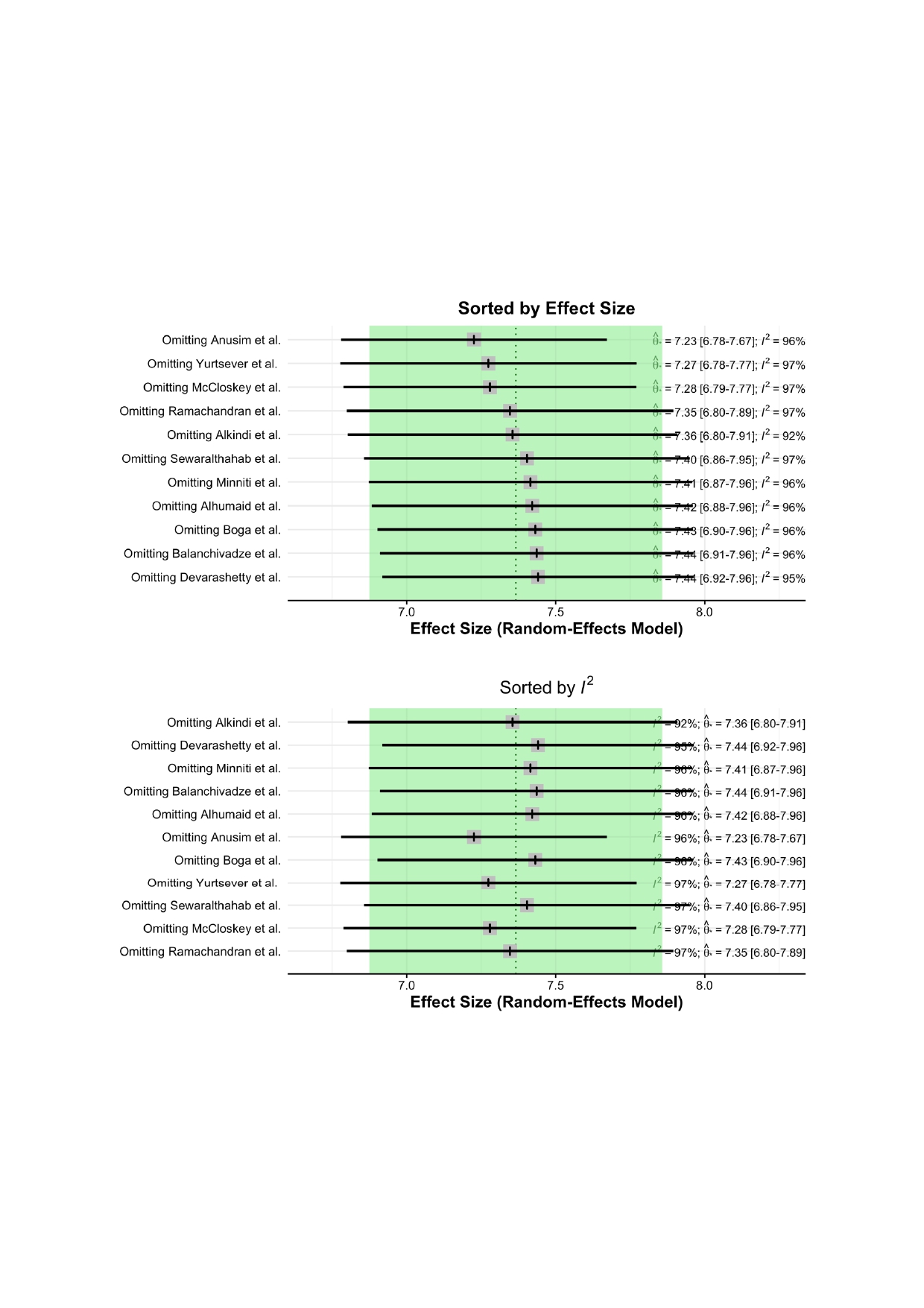
**Supplementary Figure 3.** Sensitivity analysis of serum ferritin on admission.
